# Qualitative ultrasonography scale of the intensity of local twitch response during dry needling and its association with modified joint range of motion: a cross-sectional study

**DOI:** 10.1186/s12891-021-04592-z

**Published:** 2021-09-14

**Authors:** Carlos Cruz-Montecinos, Mauricio Cerda, Pablo Becerra, Claudio Tapia, Rodrigo Núñez-Cortés, Rodrigo Latorre-García, Sandro R. Freitas, Antonio Cuesta-Vargas

**Affiliations:** 1 Department of Physical Therapy, Faculty of Medicine, University of Chile, Santiago, Chile; 2 Laboratory of Biomechanics and Kinesiology, San José Hospital, Santiago, Chile; 3 Integrative Biology Program, Institute of Biomedical Sciences, Faculty of Medicine, Universidad de Chile, Santiago, Chile; 4 Center for Medical Informatics and Telemedicine, Faculty of Medicine, Universidad de Chile, Santiago, Chile; 5 Biomedical Neuroscience Institute, Santiago, Chile; 6 Neuromuscular Research Lab, CIPER, Faculty of Human Kinetics, University of Lisbon, Lisbon, Portugal; 7 Departamento de Fisioterapia, Andalucía Tech, Catedra de Fisioterapia y Discapacidad, Instituto de Investigación Biomedica de Málaga (IBIMA), Clinimetria (F-14), Universidad de Málaga, Málaga, Spain; 8 School of Clinical Science, Faculty of Health at Queensland University Technology, QLD Brisbane, Australia

**Keywords:** Ultrasound, Local Twitch Response, Dry Needling, Range of Motion, Trigger Points

## Abstract

**Background:**

The relevance of local twitch response (LTR) during dry needling technique (DNT) is controversial, and it is questioned whether LTR is necessary for successful outcomes. Furthermore, because the LTR during the deep DNT may be evoked with different intensities, it is unknown whether the magnitude of LTR intensity is associated with optimal clinical results, especially concerning to the effects of joint maximal range of motion (ROM). This study aimed to (i) determine whether visual inspections can quantify the LTR intensity during the DNT through a qualitative ultrasonography scale of LTR intensity (US-LTR scale), and (ii) assess the differences of US-LTR scale associated with changes in the maximal joint ROM.

**Methods:**

Using a cross-sectional design, seven asymptomatic individuals were treated with DNT in the latent myofascial trigger point in both medial gastrocnemius muscles. During DNT, three consecutive LTRs were collected. The US-LTR scale was used to classify the LTRs into strong, medium, and weak intensities. The categories of US-LTR were differentiated by the velocity of LTRs using the optical flow algorithm. ROM changes in ankle dorsiflexion and knee extension were assessed before and immediately after DNT.

**Results:**

The US-LTR scale showed the third LTR was significantly smaller than the first one (*p* < 0.05). A significant difference in velocity was observed between US-LTR categories (*p* < 0.001). A significant difference in the ROM was observed between the strong and weak-medium intensity (*p* < 0.05).

**Conclusions:**

The present findings suggest that the LTR intensity can be assessed using a qualitative US-LTR scale, and the effects of DNT on joint maximal ROM is maximized with higher LTR intensity. This study reports a novel qualitative method for LTR analysis with potential applications in research and clinical settings. However, further research is needed to achieve a broader application.

## Introduction

The Myofascial trigger points (MTrPs) have been defined as hyperirritable nodules in tense bands of skeletal muscle that exhibit motor, sensory, and autonomic components and classified as either active or latent [43]. MTrPs have been reported to be prevalent in both healthy [46] and chronic musculoskeletal pain [6, 35]. The latent MTrPs are defined as a focus of hyperirritability in a muscle taut band that is clinically associated with local twitch response and tenderness and/or referred pain upon manual examination [16], while active MTrPs are characterized by spontaneous local and referred pain [16, 21, 40].

The prevalence of latent MTrPs in the lower limb of asymptomatic people has been reported to be around 78 % [46], whereas the medial gastrocnemius has the highest prevalence (i.e. 80 %) of latent MTrPs among the triceps sural muscles [18]. In latent MTrPs, it could also involve an increase in spontaneous electrical activity, which can result in a higher H-reflex amplitude, suggesting more active muscle spindle afferents [15]. Although the latent MTrPs do not cause discomfort, they may affect the maximal joint range of motion (ROM) [2, 17, 18].

Active and latent MTrPs have been widely treated by the use of dry needling techniques (DNT) to increase ROM in healthy individuals and those with neurological and musculoskeletal disease [1, 2, 13, 14, 28]. DNT consists of partially inserting and withdrawing a needle at the MTrP site in order to obtain local twitch responses (LTRs) – i.e. a visible or palpable contraction when a needle is placed at an MTrP in the involved muscle after mechanical stimulation. The local twitch response is defined as a spinal cord reflex, resulting in a brief, involuntary muscle fibers contraction [21]. The muscle spindle excitability may be involved in the pathophysiology of latent MTrP [15]. The mechanical stimulus of DNT and the associated LTRs may modulate the excitation of muscle afferents [3, 5, 12]. The LTRs during deep DNT are assumed to be essential to the effective release of MTrPs [21, 42]. For instance, the treatment of latent MTrP (i.e., when local and referred pain is evoked with direct pressure) has been reported to decrease the resting stiffness of the muscle and improve maximal joint ROM [21, 40]. However, the relevance of LTRs during DNT is controversial, and it is questioned whether LTR is necessary for successful outcomes [31]. Furthermore, because the LTR during the deep DNT may be evoked with different intensities (i.e., the velocity of muscle fascicles shortening), it is unknown if LTR intensity is associated with optimal clinical results, especially concerning maximal joint ROM effects.

To better understand the physiology of LTR, clinical tools are necessary. For that, ultrasound-guided trigger point puncture has been proposed to detect LTR, taking into account that the detection of LTR is not always evident by visual or palpation assessment [34]. However, to the best of our knowledge no previous studies have reported LTR intensity through an ultrasound guide. This study aimed to (i) determine whether visual inspections can quantify the LTR intensity during the DNT through a qualitative ultrasonography scale of LTR intensity (US-LTR scale), and (ii) assess the differences of US-LTR scale associated with changes in the maximal joint ROM. We hypothesized that the US-LTR scale is a valid convergent tool to quantify the intensity of LTR, and a greater LTR intensity implies a greater maximal ROM after DNT.

## Methods

### Participants

Using a cross-sectional and non-probability sampling, seven healthy young men (age: 28.0 ± 3.1 years; height: 1.76 ± 0.07 m; body mass index: of 24.8 ± 2.9 kg/m^2^) were recruited from San José Hospital, Santiago Chile (employees only). Ethical authorization was obtained from the Ethics Committee of the Northern Metropolitan Health Service of Santiago, Chile. All participants agreed to participate in this study and signed an informed consent form. All methods were carried out in accordance with relevant guidelines and regulations. The study was registered in ClinicalTrials.gov (NCT02824991) and reporting adhered to the STROBE guidelines.

### Inclusion and exclusion criteria

The inclusion criteria were as follows: men between 20 and 50 years of age. The inclusion was limited to only males to control the potential modulatory role of sex hormones to muscle stretch reflex [4], as well the potential gender differences of tolerance during stretch maneuvers (see below) [25]. The exclusion criteria were as follows: body mass index > 30, history of any signs or symptoms of musculoskeletal pain in the last 6 months, pathological conditions of the vertebral column, neurological diseases, respiratory diseases, a systemic rheumatic condition, heritable disorders of connective tissue, and/or any previous abdominal surgery.

### Dry needling technique procedure

**Fig. 1:**
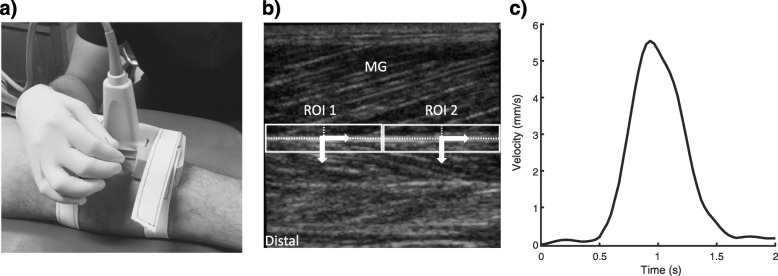
Experimental setup and data normalization. **a** Filament needle inserted into muscle trigger points while keeping the ultrasound transducer fixed. **b** Regions of interest (ROI) positioned on deep aponeurosis. Medial gastrocnemius (MG). **c** Example of signal velocity of one local twitch responses

The participants were tested in one session, with a cross-sectional study design. All measurements were made in the San José Hospital, Santiago, Chile. The participants received the DNT intervention for the medial gastrocnemius of both lower limbs (i.e., total of 14 legs), with a randomized order. Participants lay in a prone position with the ankle fixed at 90º (i.e., the angle between the lateral border of the foot and horizontal). A local examiner (CCM) ensured the maintenance of ankle and knee angle during the DNT intervention. The localization of latent MTP at the medial gastrocnemius was identified by determining a palpable and hypersensitive taut band criteria [18, 43]. The ultrasound transducer was then positioned on the MG muscle orientated according to fascicles direction close to the MTrPs using a cast composed of a thermoplastic polymer, with enough space to perform the DNT (Fig. 1A). The deep DNT was administered by a physical therapist with 3 years of experience (PB) using an acupuncture needle (0.30 × 50 mm; Huan Qiu, Suzhou, China). The ultrasound video (SonoSite Titan; Sonosite, Bothell, WA, USA) with a linear transducer (5–10 MHz) was recorded at 30 Hz and captured through an external capture device from Epiphan Systems Inc. (Ottawa, Ontario, Canada). According to the perception of the physical therapist to detect the latent MTrPs and a hypersensitive tender spot within the taut band, the filament needle was inserted into the MTrP to achieve three LTRs. During DNT, three consecutive LTRs were recollected in each leg (42 L in total). The three LTRs were also confirmed with visual register using the ultrasound by a local examiner (CCM) [34].

### Maximal joint ROM assessment

The ankle dorsiflexion and knee extension maximal ROM were assessed (with a randomized order) before and immediately after DNT intervention. The same physical therapist (PB) performed all ROM measurements, who was blinded about the intensities of LTR collected during the DNT. The ankle dorsiflexion and knee extension maximal ROM of both lower limbs were assessed using weight-bearing and active knee extension test, respectively, as an estimate of medial gastrocnemius and hamstring extensibility. A universal goniometer (Baseline, 360° marked in 1° increment; Prestige Medical, Northridge, CA, USA) was used to perform the joint ROM testing. Three measurements were performed for each test, which has shown to have an excellent inter-rater reliability for the ankle and knee maximal ROM [10].

For the active knee extension test, the participants were in a supine position with the contralateral knee extended and the hip in a neutral position and the ipsilateral hip and knee flexed to 90° [45]. A belt was placed across superior iliac spine to prevent the pelvic and lumbar movement during the testing. Each participant extended the knee to the maximum tolerable ROM. From there, two lines were draw so the goniometer arms could be aligned. The first was drawn to the greater trochanter, and the other was drawn to the apex of the lateral malleolus [19]. For this, 0° was considered the knee in the full extension position. The active knee extension test using goniometry has been reported to have an excellent inter-rater reliability (ICC = 0.91, with 95 % confidence interval of (0.87–0.93), and minimal detectable difference of 8 ° [27].

For the weight-bearing ankle dorsiflexion ROM test, the participants were instructed to keep the knee of the tested leg extended and then to maximally flex their tested ankle while keeping their heel on the floor. The weight-bearing test using an standard goniometer has been reported to be a valid test to assess the ankle dorsiflexion maximal ROM [38, 41]. A verbal feedback was given to prevent the excessive pronation of the foot during the maneuver. The stationary arm of the goniometer was pointed to the proximal head of the fibula, and the moving arm was placed parallel with the lateral border of the foot. This test has been reported to have excellent inter-rater reliability (ICC = 0.96 with, 95 % confidence interval of 0.91–0.99), and minimal detectable difference of 4.7 ° [22, 32].

### Data processing and classification

Sonographic data was processed using Matlab® scripts (v2014, Mathworks Inc., Natick, MA, USA). The LTRs were examined offline by a physical therapist (CCM) with 5 years of experience in muscle ultrasound imaging. The Camtasia software (Tech-Smith Corp, Okemos, MI, USA) was used to select the three LTRs for later analysis. A visual criterion was established to differentiate muscle movement due to the DNT and LTRs, considering the vertical and horizontal movement in the deep aponeurosis. It should be noted that when the dry needle is inserted into the muscle, only a vertical displacement is produced.

#### Automatic tracking

The deep aponeurosis was tracked by the Lucas–Kanade optical flow algorithm with affine optic flow extension [23]. The LTR intensity was based on velocity of the deep aponeurosis motion (i.e., Euclidian distance / time between frames). It should be noted that the magnitude of muscle fascicles shortening evoked by twitch response associates to the deep aponeurosis motion [24]. The deep aponeurosis motion during LTR was considering as gold standard. The Matlab algorithm used here, by David Young, is available in Matlab Central (https://la.mathworks.com). The parameters used were a sigma value of 1 and a sample step of 1. Two regions of interest with a standard size for width (based on the half of the US image width) and adapted size por height depended on the thickness of the deep aponeurosis (four times of deep aponeurosis thickness), were manually selected at the center of the echogram map by including deep aponeurosis (i.e. hyperechoic line) and its surrounding muscle borders (Fig. 1B), to determine the peak velocity during the LTR. The instantaneous root-mean-square via convolution with a window of 500 ms was used to estimate the velocity (Fig. 1C). The root-mean-square algorithm used here by Scott McKinney is available in Matlab Central (https://la.mathworks.com). The maximal velocity between both regions of interest was determined and considered for the statistical analysis.

#### US-LTR scale

After deep aponeurosis motion processing, two raters with low experience (novices) in ultrasound (RNC and CT) classified LTR in three intensity categories: weak, medium, and strong. For this purpose, one rater with high experience (expert) in ultrasound (CCM) gave an explanatory session (2 hours) to the novice raters concerning the visual interpretation (in b-mode recordings) of LTR with different intensities. The instruction was focused on the velocity of the deep aponeurosis. Three LTR were presented with deep aponeurosis motion velocities of 2.8 mm/s (i.e., weak), 3.3 mm/s (i.e., medium), and 5.4 mm/s (i.e., strong). The qualitative scale (US-LTR) was practiced by the novices in three videos prior to the analysis. The high and low experienced raters were blinded to the maximal ROM values after DNT, and the values of maximal velocity of LTR.

### Statistical analysis

All data were analyzed with the SPSS software (v. 22.00 for Windows, IL, USA). The normality of data distribution was evaluated using the Shapiro-Wilk test. To compare the intensities of the three consecutive LTRs recorded through the US-LTR scale, the Friedman test and multiple comparisons with Dunn’s correction were used. The agreement of the US-LTR scale between the expert and the two novices, and between novices was evaluated using the Kappa statistic; and the agreement result was interpreted as none (≤ 0), none or weak agreement (0.01–0.20), fair (0.21–0.40), moderate (0.41–0.60), substantial (0.61–0.80), and almost perfect (0.81–1.00) [26]. To compare the velocities between the three consecutive LTRs and the three categories of the US-LTR scale (i.e., strong, medium, and weak intensities), the mixed-effect analysis and multiple comparisons with Bonferroni corrections were used. To compare the pre-post effect of DNT on maximal ROM, the paired t-test was used. To compare the differences in the increased maximal ROM between the weak-medium and strong intensities of the first LTR (assessed by the expert), the unpaired t-test was used. The decision to include only the first LTR was made based on the assumption that the intensity of the second and third LTR may decrease as compared with the initial LTR [3]. The weak-medium intensities of the first LTR were collapsed, in order to compare the same number of legs between groups (week-medium: *n* = 7 legs and strong: *n* = 7 legs). Unpaired testing was conducted to compare groups (week-medium vs. strong) because only three participants were members of both groups.

A *p*-value of < 0.05 was considered statistically significant. The data was expressed as mean and 95 % confidence interval unless stated otherwise. For parametric comparison, the effect sizes was established by calculating Cohen’s *d* (*d* > 0.2, *d* > 0.5, or *d* > 0.8) to indicate small, moderate, or large effects, respectively [29]. For non-parametric comparison, the effect size was calculated through an *r* conversion of the z-score (*r* > 0.1, *r* > 0.3, or *r* > 0.5) to indicate small, moderate, or large effects, respectively [30].

The sample size needed for this study was calculating using GPower software, version 3.1.9.2 (Universität Düsseldorf, Germany). Considering a previous reported large effect size on maximal ROM after treatment of latent MTrPs [17], seven participants were determined enough to reach a p-value of < 0.05 and β of 0.20.

## Results

### Assessment through US-LTR scale

**Fig. 2:**
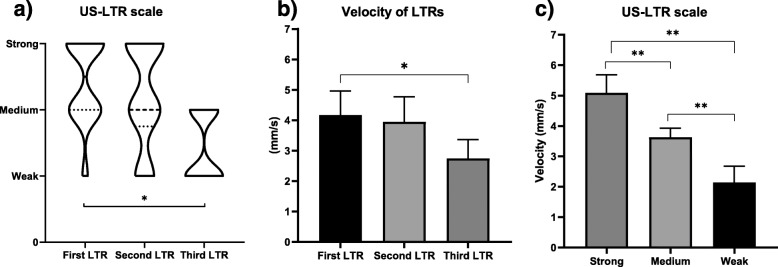
The local twitch response (LTR) intensities. **a** The qualitative analysis using ultrasonography scale of LTR intensity (US-LTR) among the three consecutive LTRs. **b** The quantitative analysis using automatic tracking among the three consecutive LTRs. **c** LTR velocities between the three categories of the US-LTR scale. Strong (*n* = 13), medium (*n* = 16), and weak (*n* = 13). Values are means and 95 % confidence intervals. *p < 0.05 and **p < 0.001

The qualitative analysis of intensities using the US-LTR scale showed a significant difference between the consecutive three LTRs (p = 0.003) (Fig. 2A). The post-hoc analysis showed that the third LTR was significantly smaller than the first one (*p* = 0.008; *r* = 0.8, large effect), but not between the first and second (*p* = 0.999; *r* = 0.3, small effect), and second and third LTRs (*p* = 0.113; *r* = 0.56, large effect) (Fig. 2A).

### Assessment of LTR velocity

The LTR velocity showed a significant difference between the consecutive three LTRs (*p* = 0.013) (Fig. 2B). The post-hoc analysis showed that the third LTR was significantly smaller than the first one (*p* = 0.018; *d* = 0.79, moderate effect), but not between the first and second (*p* = 0.999; *d* = 0.11, small effect), and second and third LTRs (*p* = 0.057; *r* = 0.76, moderate effect) (Fig. 2B). The tracking of LTR velocity also showed a significant difference between the three scales of the US-LTR scale (i.e., strong, medium, and weak intensities) (*p* < 0.001). The post-hoc analysis showed a significant difference and large effect size for all comparisons (*p* < 0.001; *d* > 0.8) (Fig. 2C).

### Agreement of US-LTR scale

**Fig. 3:**
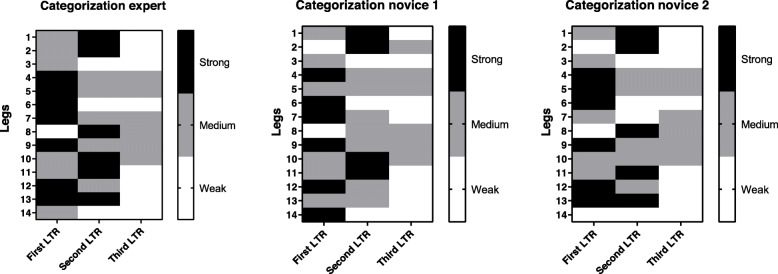
Agreement of qualitative ultrasonography scale of local twitch response (LTR) intensity (US-LTR). Hot map of US-LTR scale between expert and novices (*n* = 42 L)

Considering all LTRs recorded (42 in total), inter-rater agreement of US-LTR scale (strong, medium, and weak intensities) between the expert and the two novices was substantial (kappa: 0.720–0.80, *p* < 0.001) (Fig. 3). The agreement between the two novices was also substantial (kappa: 0.64, *p* < 0.001) (Fig. 3).

### US-LTR scale and effects in maximal ROM

**Fig. 4:**
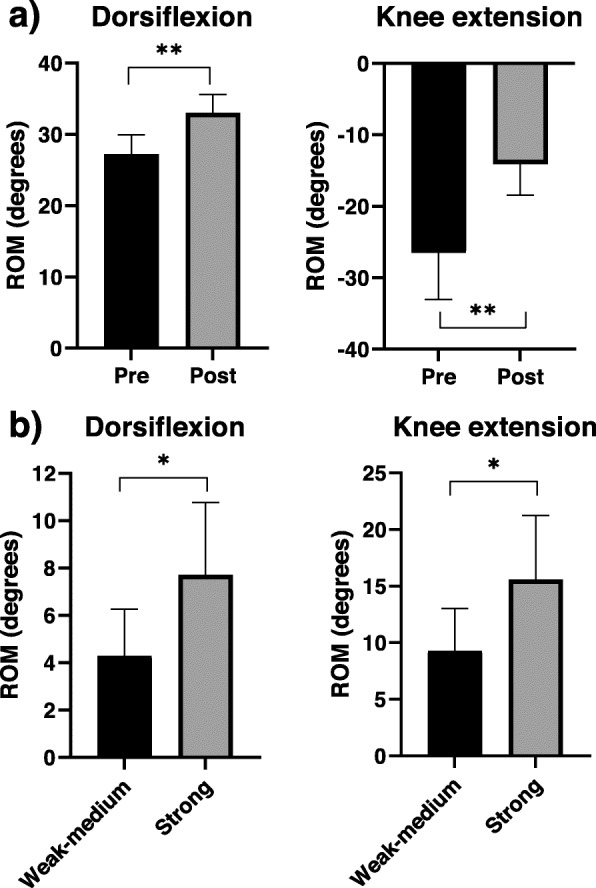
The ultrasonography scale of local twitch response intensity (US-LTR) and scale and effects in maximal range of motion (ROM). **a** Pre-post maximal ROM (*n* = 14 legs). **b** Differences in the ROM between the strong (*n *= 7 legs) and weak-medium (*n* = 7 legs) intensities. Values are means and 95 % confidence intervals. *p < 0.05 and **p < 0.001

Following DNT, significant changes in the maximal ROM for knee extension and dorsiflexion (*p* < 0.001) (Fig. 4A; Table 1) were observed. A significant difference in the ROM was observed between the strong and weak-medium intensities (ankle: *p* = 0.040 and knee: *p* = 0.043, respectively) (Fig. 4B; Table 2B).

**Table 1:** Pre-post maximal ROM

ROM	Pre dry needling	Post dry needling	Mean difference	*p*-value	Effect size (*d*)
Dorsiflexion	27.2 [24.5 to 30.0]	33.0 [ 30.4 to 35.6]	5.8 [4.0 to 7.6]	< 0.001 **	1.7 (large)
Knee extension	-26.5 [-33.1 to -20]	-14.1 [-18.4 to -9.7]	12.4 [ 9.0 to 15.9]	< 0.001 **	2.1 (large)

Values are expressed in degrees. Pre dry needling (*n* = 14 legs) and post dry needling (*n* = 14 legs). Values are means and 95 % confidence intervals. Maximal range of motion (ROM)

** *p* < 0.001.Cohen’s d (*d*)

**Table 2:** Differences in the ROM between LTR intensities

ROM	Week-medium	Strong	Mean difference	*p*-value	Effect size (*d*)
Dorsiflexion	4.3 [2.3 to 6.3]	7.7 [ 4.7 to 10.8]	3.4 [0.19 to 6.7]	0.040 *	1.2 (large)
Knee extension	9.3 [5.6 to 13.0]	15.6 [9.9 to 21.2]	6.3 [ 0.2 to 12.3]	0.043*	1.1 (large)

Values are expressed in degrees. Week-medium (legs = 7) and strong (legs = 7). Values are means and 95 % confidence intervals. Maximal range of motion (ROM)

*p < 0.05. Cohen’s d (*d*)

## Discussion

The aim of this report was to determine whether the LTR intensity during DNT can be quantified through visual inspection using a qualitative ultrasonography scale of LTR intensity (US-LTR scale), as well as to assess the differences between the intensities of LTRs associated with changes in maximal joint ROM. The US-LTR scale and automatic tracking of the deep aponeurosis (gold standard) showed a significant difference in LTR intensity between the first and third LTR. Moreover, the findings of this study show good agreement with the qualitative US-LTR scale between the expert in ultrasound and novices. Furthermore, this study indicated that the intensities of LTRs (i.e., strong versus weak-medium intensity) may involve different magnitudes of maximal ROM changes after DNT interventions. The present result supports the hypothesis that the US-LTR scale is a valid tool to quantify the intensity of LTR, and a greater LTR intensity implies a greater maximal ROM after DNT.

The US-LTR scale was sensitive enough to identify a decrease in intensity between the first and third LTR. The decrease in LTR intensities observed in this study agrees with a recent study where the LTR intensities were assessed with surface electromyography during the application of DNT over latent MTrPs in the medial gastrocnemius [3]. The decreased in LTR intensities during the DNT observed in our study may be explained by electrophysiological mechanisms [3, 5]. In latent MTrPs, muscle spindle afferents may be involved in the pathophysiology of MTrPs [15]. The mechanical stimulus of DNT and the associated LTRs may modulate the excitation of muscle afferents to spinal cord motor-neurons [3, 5, 12]. Likewise, the relaxation of MTrPs could be progressive and linked to LTRs, which would explain the highest intensity occurring for the first LTR and a decreased intensity in the third LTR.

We observed a significant difference in the maximal joint ROM between the strong and weak-medium intensities. Improved joint flexibility following DNT has been previously reported [1, 2], showing a greater impact on flexibility than a placebo [36]. The observed changes in joint flexibility could be associated with a relaxation of the MTrPs. The LTR has been associated with inhibitory factors in spontaneous electrical activity during DN [5], as well with decreased muscle stiffness [1, 2]. The remote effect of DNT observed in this study could be explained by H-reflex modulation and myofascial continuity between the gastrocnemius and hamstring, as has been observed in *in vivo* models [7, 44]. The qualitative assessment of the intensity of LTRs using the US-LTR scale can be used to predict the changes in ROM after DNT, providing the possibility of incorporating a more specific dose-response strategy in DNT protocols.

Regarding the clinical implications of these results, ultrasound imaging technology is currently increasing in use among health professionals to assess muscle function and puncture guidance [11, 39]. Because the LTR during the deep DNT may be evoked with different intensities, it is unknown whether the magnitude of LTR intensity is associated with optimal clinical results. The US-LTR scale is a potential tool to evaluate the LTR intensities in real-time during DNT interventions. Also, the LTR intensity is not usually reported in DNT protocols and both the number of LTRs and their intensities need to be reported in future studies.

This study has several limitations. First, the joint torque and electromyography were not assessed. Therefore, it is unclear the time related of LTR with proprioceptive afferents reflex and the motor unit activity of the investigated muscles. Second, the effect of DNT on maximal ROM was not counteracted with a placebo group (i.e., sham dry needling) [8], which should be considered in future studies. Third, we only measured healthy mean, so these results cannot be extrapolated to females. For this, future studies are needed to investigate gender differences in LTR intensity and its effect on the maximal ROM. Fourth, the US-LTR scale was validated in a pennate muscle. Future studies are needed in parallel muscles (e.g., fusiform). For this, the change in muscle thickness might be the outcome of the qualitative scale. Fifth, the approach used to assess LTR velocities assume a two-dimensional behavior of LTR when three-dimensional shape changes emerge from muscle contraction [9, 33]. Sixth, the LTRs were recorded at 30 Hz. A faster recording (i.e., ultrafast ultrasound, with > 1000 Hz) may improve the accuracy of the assessment of LTR velocity [20, 37].

Finally, future randomized clinical trials should investigate the clinical implications of LTR intensities during the dry-needling intervention in musculoskeletal and neurological diseases.

## Conclusions

The present findings suggest that the LTR intensity can be assessed using a qualitative US-LTR scale, and a greater LTR intensity implies a greater ROM post dry needling intervention. This study reports a novel qualitative method for LTR analysis with potential applications in research and clinical settings. However, further research is needed to achieve a broader application.

## Data Availability

The datasets analyzed during the current study are available from the corresponding author on reasonable request.
